# Family Composition and Stability for Orphans: A Longitudinal Study of Well-Being in 5 Low- and Middle-Income Countries

**DOI:** 10.3389/ijph.2021.1604057

**Published:** 2021-12-21

**Authors:** Christine L. Gray, Kathryn Whetten, Julie L. Daniels, Michael G. Hudgens, Audrey E. Pettifor, Amy M. Hobbie, Nathan M. Thielman, Misganaw E. Dubie, Dafrosa Itemba, Ira Madan, Vanroth Vann, Augustine I. Wasonga, Rachel Manongi, Jan Ostermann, Rachel A. Whetten, Brian W. Pence

**Affiliations:** ^1^Department of Epidemiology, Gillings School of Global Public Health, University of North Carolina at Chapel Hill, Chapel Hill, NC, United States; ^2^Center for Health Policy and Inequalities Research, Duke Global Health Institute, Duke University, Durham, NC, United States; ^3^Sanford School of Public Policy, Duke University, Durham, NC, United States; ^4^Department of Biostatistics, Gillings School of Global Public Health, University of North Carolina at Chapel Hill, Chapel Hill, NC, United States; ^5^Division of Infectious Diseases, Department of Medicine, School of Medicine, Duke University, Durham, NC, United States; ^6^Stand for Vulnerable Organization, Addis Ababa, Ethiopia; ^7^Tanzania Women Research Foundation, Moshi, Tanzania; ^8^Sahara Centre for Residential Care and Rehabilitation, New Delhi, India; ^9^Development for Cambodian Children, Battambang, Cambodia; ^10^Ace Africa-Kenya, Bungoma, Kenya; ^11^Kilimanjaro Christian Medical Centre, Moshi, Tanzania; ^12^Department of Health Services Policy and Management, Arnold School of Public Health, University of South Carolina, Columbia, SC, United States

**Keywords:** cognition, orphans, LMIC, family composition, stability, well-being, abuse

## Abstract

**Objectives:** Many orphaned children in low- and middle-income countries live with family. Yet, their household composition and its stability are not well-characterized, nor is impact of stability on longer-term outcomes.

**Methods:** We used the longitudinal, multi-country Positive Outcomes for Orphans cohort to describe adult family living with orphans. Stability was measured by changes in presence of six familial relations over time, and related to three outcomes: 1) incident abuse, 2) cognitive functioning, 3) emotional difficulties. Associations were estimated using generalized linear models fit with generalized estimating equations. For abuse, Poisson regression estimated risk ratios. For continuous scores of cognitive functioning and emotional difficulties, linear models estimated mean differences (MDs) with 95% confidence intervals.

**Results:** Among 1,359 orphans, 53–61% reported living with their mother each year; 7–13% with father; nearly 60% reported ≥1 change in composition over follow-up. Compared to 0 changes, difficulties increased with 1 change [MD: 0.23 (−0.33, 0.79)], 2 changes [MD: 0.57 (0.00, 1.16)] and ≥3 changes [MD: 0.73 (0.18, 1.29)]. No associations were found with abuse or cognitive functioning.

**Conclusion:** Orphan well-being may be improved through supports stabilizing household composition or targeting emotional resilience.

## Introduction

In 2016, the world’s orphan population was estimated to be 140 million [1]. Millions more children and adolescents, called “separated,” have been abandoned by or permanently disconnected from parents because of poverty, war, or other crises. Low- and middle-income countries (LMICs) have disproportionately greater numbers of orphaned and separated children (OSC), in part due to the HIV and AIDS epidemic and its coupling with poverty and civil conflicts [2].

Ensuring OSC receive quality care is a global public health issue because it impacts their short- and long-term physical and mental health, and in turn their functioning as adults. The Sustainable Development Goals (SDG) specifically target promotion of mental health and wellbeing, as part of the goal to reduce mortality from noncommunicable diseases (SDG 3.4) [3]. OSC are particularly susceptible to adverse outcomes, including food insecurity [4], stigma [5], emotional and cognitive deprivation [6, 7], trauma [8, 9], and HIV-related risk behaviors [10]. These vulnerabilities, compounded by disproportionate numbers, make caring for OSC from childhood through adolescence particularly challenging in LMICs. OSC can live in different settings, including residential care (i.e., orphanages, group homes), family-based care (i.e., in a caretaker’s family home), on their own (i.e., child-headed households), or on the street. Residential care is provided by non-relatives; family-based care may involve non-relatives or relatives, including the remaining parent.

Family-based care has been promoted as the universally ideal option [11, 12], in part because it is thought to provide a more stable caregiving environment. Stability in turn may impact overall well-being, through facilitation of cognitive growth and protection against abuse and emotional difficulties. Indeed, studies in the United States (U.S.) suggest familial stability is critical for development; transitions in familial composition have been associated with worse behavioral outcomes [13–15]. Similar studies in LMICs are lacking. Understanding the family-based environment of OSC in LMICs is essential for identifying the supports requisite for long-term functioning and well-being. A critical first step is quantifying how many family-based OSC are living with a remaining parent or relative, the extent to which that adult family composition is stable over time, and how instability in that composition may relate to adolescent well-being.

The Positive Outcomes for Orphans study (POFO) is a longitudinal OSC cohort from six culturally and geographically diverse sites in five LMICS. We focus on 1,359 OSC in family-based care to characterize the adult family composition in their household, and the extent to which changes in that composition are associated with three outcomes that may serve as markers of longer-term trajectories: incident abuse, changes in cognitive functioning, and emotional well-being.

## Methods

### Study Population

We used data from the POFO study, a longitudinal cohort of OSC randomly sampled from both family-based care and residential care in six sites: Battambang, Cambodia; Addis Ababa, Ethiopia; Hyderabad and Nagaland, India; Bungoma, Kenya; Kilimanjaro Region, Tanzania. At baseline enrollment (May 2006–February 2008, depending on site), children were ages 6–12. We used an analytic baseline starting 12 months after enrollment because the exposure of interest, familial adult household composition, was not measured until 12-months follow-up. For the measures in this study, OSC were interviewed approximately annually, totaling seven observation rounds (6 years).

The sampling design has been described elsewhere [16]. Briefly, family-based children were selected by randomly sampling 50 clusters identified using administrative and geographic boundaries; five children in each cluster were randomly sampled using available lists or house-to-house census until five age-eligible OSC were identified. A comparison group of non-OSC (1 per cluster) was sampled in the same manner to provide context for the experience of non-OSC from the same regions; we included parallel analyses on non-OSC. The sampling frame was not always known for the family-based children and thus sampling probabilities were not available to construct sampling weights [16]. Residentially-based OSC (not reported in this study on family-based care) were sampled separately.

### Exposure: Change in the Familial Adult Household Composition

The exposure for our analyses is cumulative change in the familial adult household composition. Specifically, we measured whether there was a change (gain or loss) in six key adult relations in the household since the last interview, and quantified the accumulation of those changes over time. We used questions asking whether the study participant was living with each of the following adult relations presumed to be part of the caregiving structure: father, mother, stepfather, stepmother, grandfather, grandmother. The questions ask, “Of the adults and children living in your household, how many are your … (mother, father, etc).”

We calculated the exposure in three steps. First, for each of the six identified adult relations, we defined “change” as reporting differently than the last interview. To assess overall stability of the household composition, we treated both gains and losses as “changes.” For example, if a child reported living with their father at time X but not at time X+1, then we coded “change in father” as a “1” at time X+1. Second, we created a variable for “total changes” that summarized how many changes were experienced at a given time point, for a maximum of six changes within a given round if a change occurred with each relation that round. Across rounds, a change can occur more than once with the same relation. For example, if a father moves out, and then later moves back in, then that would constitute two distinct changes (counted at different rounds). Third, we created a “cumulative change” variable that accumulated over time to reflect the total number of changes in the familial adult composition the child had experienced up to that point across all six adult relations. We treated each relation equally, i.e., we did not weight, for example, parents over grandparents. To our knowledge, there is no established metric for doing so.

### Orphan Well-Being Outcomes

Three outcomes were identified as central to the overall well-being of OSC: incident abuse, emotional well-being, and cognitive functioning.

Incident abuse was defined using self-reported responses to the Life Events Checklist (LEC), a 17-item inventory of traumatic experiences that is used in diverse cultural settings [17, 18]. The LEC was only administered to children if they were at least 10 years old, based on pilot testing and IRB recommendations. We used the four questions asking whether the participant experienced 1) unwanted touching of private sexual parts, 2) rape or molestation, 3) being hit, kicked, or beaten at home, or 4) being hit, kicked or beaten by other children in the past year. Endorsement of experiencing any of these four events in the past year was coded as “1” for this binary outcome.

Emotional well-being was defined using self-reported responses to the Strengths and Difficulties Questionnaire (SDQ). Like the LEC, the SDQ was only administered to children who were at least 10. We focused on the 20 “difficulties” items, consistent with common usage of the SDQ [19, 20], and prior studies in this population [16, 21]. These 20 items include questions such as “I am restless; I cannot sit still” and “I have many fears; I am easily scared.” Each item was evaluated on a three-point scale of “Not,” “Somewhat” or “Certainly” true, and coded as 0, 1, or 2, respectively; total difficulties scores range from 0 to 40. The final score was a summation of values across items. A higher score indicates worse emotional well-being for this continuous outcome.

Cognitive functioning was defined using the Market List, a culturally-adapted, site-specific version of the California Verbal Learning Test (CVLT) [22] in which memory and verbal learning are assessed through recitation of items familiar to participants from their local markets. This measure captures short-term memory and attention, and was validated in prior analyses as a measure of learning that could be assessed with fidelity in LMICs [23]. Interviewers read a list of 15 locally familiar market items to the participant, and tested the participant’s ability to recall the items. To preserve consistency with the original measure, items were identified from categories in the CVLT (things a child eats, wears, and plays with). We scored the Market List by averaging the number of items recalled in three trials of an interviewer reading and a participant repeating the 15-item list; average scores range from 0 to 15. We used change since analytic baseline as the outcome to account for baseline functioning. A higher score indicates better cognitive functioning for this continuous outcome.

### Covariates

In each analysis, we controlled for gender, age, study site (country), time, parental death status, and history of abuse. Age in years was calculated by subtracting the child’s date of birth from the interview date. Time was measured in 1-year units corresponding with annual rounds of data collection. While orphaning is a defining characteristic of inclusion in the family-based OSC group, the status of parental deaths can vary. The child may be a single orphan (one parent remaining) or may have been permanently abandoned or separated (and neither parent is known to be deceased). Therefore, we controlled for parental status (neither dead (referent), single orphan, double orphan). Because over half of OSC in this population have experienced abuse by age 13 [9], we controlled for history of abuse. This was defined by endorsement of any of the four LEC abuse questions, but was not subject to the past-year requirement. We coded history of abuse as a binary variable where “1” indicated ever and “0” indicated never experiencing abuse prior to each interview.

### Statistical Analyses

The exposure, cumulative number of changes over time, was categorized into four categories for statistical analyses: 0 changes (referent), 1 change, 2 changes, and 3 or more changes. Associations between the exposure and each of the three outcomes were assessed using generalized linear models fit using generalized estimating equations (GEEs) to account for repeated measures [24]. An autoregressive correlation structure was assumed; robust standard error estimates were used for inference.

Associations with incident abuse were estimated using Poisson regression and reported as risk ratios (RRs) with 95% confidence intervals (CIs) [25]. Because incident abuse is defined as past-year, the exposure was lagged one round to ensure it was measured prior to the interval for which abuse was reported.

Associations between the exposure and both cognitive functioning and emotional well-being were estimated using linear models and reported as mean differences with 95% CIs. The exposure was not lagged for the cognitive functioning or emotional well-being models because these outcomes measured the child’s state at the time of survey administration; any reported household changes occurred prior to outcome measurement.

Missing data, due either to incomplete observations or missed visits, were assumed missing at random (MAR), conditional on observed data [26]. Multiple imputation (MI) was used to account for missing data. The Markov Chain Monte Carlo (MCMC) data augmentation algorithm in Stata was used to create ten imputation (complete) data sets for analysis [27]. Point estimates and standard error estimates from the ten completed data sets were combined using Rubin’s method [28, 29].

Analyses were conducted using Stata 14 [30].

### Ethical Considerations

The POFO study was approved annually by Institutional Review Boards at the PI’s university and each study site. Caregiver consent and child assent were obtained from participants; at age 18, children were consented as adults.

## Results

Among the 1,359 family-based OSC in this study, approximately half (52%) were male, and the overall distribution across the six study sites was similar, ranging from 14 to 18% per site (Table 1). More than half (56%) had a deceased father, 16% had a deceased mother, and 17% had both parents deceased. Just under half (44%) were younger than 10 at the analytic baseline. The distributions in the 271 non-OSC were similar on these baseline characteristics (Table 1).

**TABLE 1:** Baseline characteristics of family-based orphans and non-orphans in the study, Positive Outcomes for Orphans study, Sub-Saharan Africa and South and Southeast Asia, 2006–2015.

	Family-based orphans (N = 1,359)		Non-orphans (N = 271)	
Characteristic	N	%	N	%
Site				
Cambodia	238	18	48	18
Ethiopia	227	17	40	15
Hyderabad, India	250	18	50	18
Kenya	193	14	40	15
Nagaland, India	219	16	49	18
Tanzania	232	17	44	16
Gender				
Male	713	52	138	51
Female	646	48	133	49
Age at analysis baseline				
7	171	13	39	14
8	211	16	47	17
9	211	16	45	17
10	218	16	66	24
11	255	19	45	17
12	201	15	20	7
13	92	7	9	3
Orphan Type				
No parents deceased^1^	144	11	N/A	
Paternal orphan	763	56	N/A	
Maternal orphan	220	16	N/A	
Double orphan	232	17	N/A	

1 Children who were abandoned by or separated from a parent due to war or other crises with no expectation of reunion are classified as orphans.

### Familial Adult Household Composition

Among the 6 adult relations, mother was the most commonly present member of the household (Table 2). At any given time point, 53–61% of children reported living with their mother. Presence of a grandmother was also common; at least 25% reported living with a grandmother at each round. Fewer reported living with a grandfather (10–21%) and still fewer reported living with their father (8–13%). Living with stepparents was infrequently reported, less than 5% in most rounds. As expected, most non-OSC were living with both their father (79–95%) and mother (87–95%); up to 29% reported a grandmother and up to 20% reported a grandfather during follow-up.

**TABLE 2:** Percentage of orphans and non-orphans with each type of adult relation in their household, at each time point, Positive Outcomes for Orphans study, Sub-Saharan Africa and South and Southeast Asia, 2006–2015.

Orphans	Father	Mother	Step-father	Step-mother	Grand-father	Grand-mother
Years since analytic baseline	%	%	%	%	%	%
0	10.5%	53.2%	1.8%	2.5%	13.1%	28.2%
1	7.8%	61.2%	2.4%	3.6%	10.3%	24.7%
2	7.4%	54.1%	2.7%	2.4%	12.6%	28.5%
3	12.9%	54.5%	9.3%	2.9%	21.4%	45.3%
4	10.1%	59.4%	3.8%	3.4%	15.3%	32.6%
5	9.0%	58.9%	6.6%	4.6%	14.2%	30.0%
6	10.1%	58.3%	4.6%	4.0%	13.7%	27.8%

**Non-orphans**	**Father**	**Mother**	**Step-father**	**Step-mother**	**Grand-father**	**Grand-mother**
Years since analytic baseline	%	%	%	%	%	%
0	86.3%	91.9%	0.0%	0.4%	4.4%	12.2%
1	94.6%	94.0%	0.0%	0.0%	5.4%	14.5%
2	91.8%	94.6%	0.8%	1.6%	3.1%	12.5%
3	79.3%	84.7%	2.9%	2.8%	19.7%	29.2%
4	83.9%	90.3%	1.7%	1.7%	10.0%	27.2%
5	81.6%	86.5%	3.9%	3.4%	9.2%	22.1%
6	79.3%	88.9%	2.0%	6.0%	9.6%	23.5%

### Changes in the Familial Adult Household Composition

**FIGURE 1:**
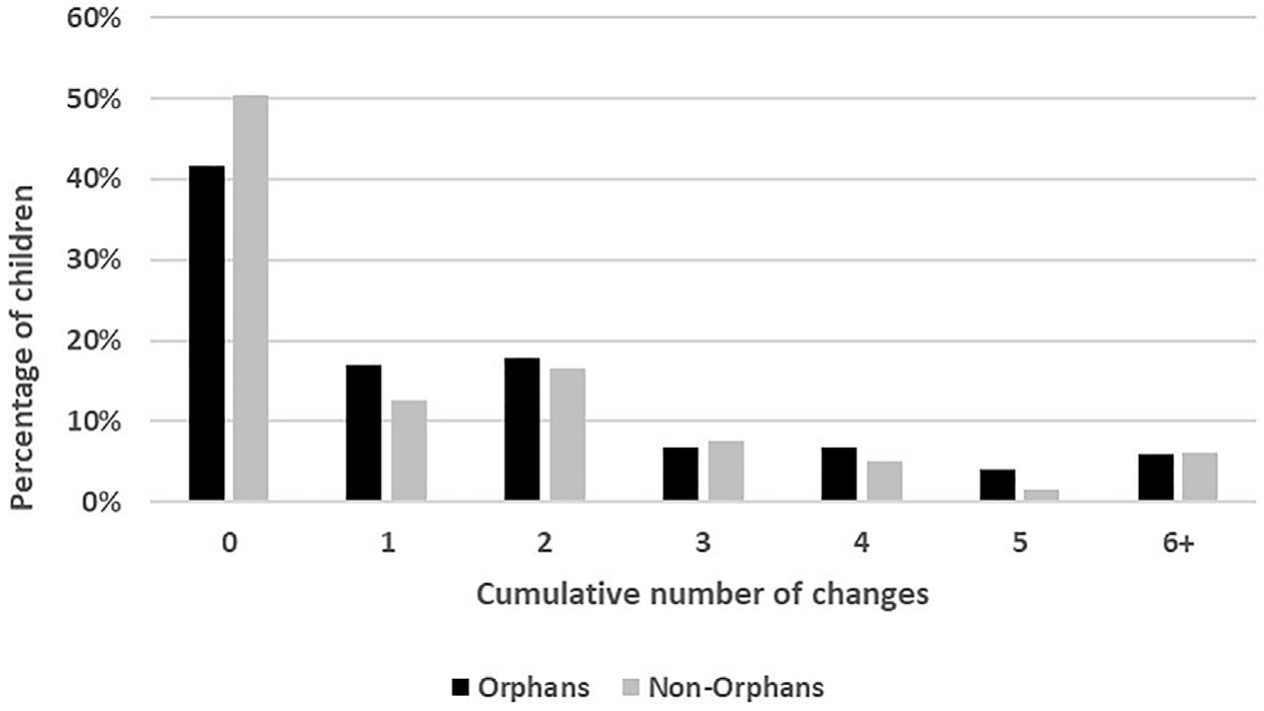
Cumulative changes experienced over follow-up, orphans and non-orphans, Positive Outcomes for Orphans study, Sub-Saharan Africa and South and Southeast Asia, 2006–2015.

Approximately 60% of OSC experienced at least one change in the adult household composition over 6 years of follow-up (Figure 1). The number of changes ranged from 0 to 11, with a mean of 1.6 and median of 1; nearly 10% experienced at least five changes in the familial adults present in the household. Non-OSC experienced a similar range (0–10), but somewhat smaller mean (1.4) and median (0); approximately 7% experienced at least five changes.

### Distribution of Outcomes

Among OSC, the average past-year incidence of abuse across all time points was 13%. The total difficulties score ranged from 0 to 31, with a mean of 8.0 and median of 7. The change in cognitive functioning score ranged from −9.7 to 10.7, with a mean of 1.3 and median of 1. Outcomes were similar but slightly better among non-OSC. The continuous outcomes (total difficulties and cognitive functioning) were normally distributed in both OSC and non-OSC populations.

### Associations With Well-Being Outcomes

Among OSC, having 2 changes or ≥3 cumulative changes over follow-up in the adult household composition was statistically significantly associated with greater total difficulties, and there appeared to be a dose-response with increasing numbers of changes and emotional difficulties (Table 3). Cumulative changes were not associated with change in cognitive functioning; estimates showed very slight decline in cognitive score but were close to the null. Having one or two changes was associated with slight decrease in relative risk of incident abuse, but 2 or more changes was associated with slightly increased risk of abuse; associations were close to null. For non-OSC, overall trends in total difficulties and incident abuse were similar, but having more than 2 changes over follow-up was slightly associated with increased (rather than decreased) cognitive functioning scores. Analyses with interaction terms between the exposure and gender did not indicate evidence of heterogeneity by gender for any of the outcomes.

**TABLE 3:** Associations between cumulative changes in adult household caregiving structure and well-being outcomes, Positive Outcomes for Orphans study, Sub-Saharan Africa and South and Southeast Asia, 2006–2015.

Orphans	Total difficulties^1^ (N = 1,356)		Cognitive functioning^2^ (N = 1,359)		Incident abuse^3^ (N = 1,356)	
Exposure	Mean difference, 95% CI		Mean difference, 95% CI		RR, 95% CI	
0 changes	Ref		Ref		Ref	
1 change	0.23	(−0.33, 0.79)	−0.07	(−0.40, 0.27)	0.93	(0.57, 1.53)
2 changes	0.57	(0.00, 1.16)	−0.04	(−0.45, 0.37)	0.92	(0.57, 1.49)
≥3 changes	0.73	(0.18, 1.29)	−0.14	(−0.59, 0.30)	1.07	(0.75, 1.54)

**Non-Orphans**	**Total Difficulties (N = 271)**		**Cognitive Functioning (N = 271)**		**Incident abuse (N = 271)**	
Exposure	mean difference, 95% CI		mean difference, 95% CI		RR, 95% CI	
0 changes	ref		ref		ref	
1 change	0.77	(−0.52, 2.06)	−0.16	(−1.03, 0.72)	1.07	(0.36, 3.12)
2 changes	0.14	(−1.05, 1.33)	0.32	(−0.52, 1.17)	0.38	(0.06, 2.36)
≥3 changes	0.89	(−0.38, 2.16)	0.53	(−0.36, 1.43)	2.40	(0.90, 6.37)

1 Total difficulties is a continuous score ranging from 0 to 40, where a higher score indicates worse emotional well-being.

2 Cognitive functioning is a continuous measure ranging from 0 to 15, where a lower score indicates lower functioning.

3 Incident abuse is a binary indicator of past-year experience of physical or sexual abuse.

RR, risk ratio; CI, confidence interval.

## Discussion

We characterized the familial adult household composition of family-based OSC in five LMICs. We further assessed the extent to which that composition changes over time and how those changes impact multiple well-being outcomes. Importantly, we found that most family-based OSC experienced changes in presence of familial adults in their household during follow-up, and those changes were associated with increased emotional difficulties. The cumulative number of changes over follow-up were not associated with incident abuse or changes in cognitive functioning.

For comparison, we also assessed a small group of non-OSC sampled from the same regions. In general, the adult family compositions for non-OSC were somewhat more stable over time, i.e., they experienced slightly fewer changes. The impacts of those changes were similar for total difficulties and incident abuse. The direction of association for change in cognitive functioning in non-OSC was opposite that of OSC for 2 and ≥3 changes. However, the associations were still null, and less precise, and thus cannot be interpreted as meaningfully different from the associations in the OSC.

To contextualize our findings, several aspects of orphan care are important to consider. Familial stability is considered important for child and adolescent well-being because it facilitates healthy attachments; it has been studied in higher-income settings such as the US [13–15] but not in OSC in LMICs. Existing studies have shown that changes, regardless of whether an adult is entering the household (a “gain”) or leaving the household (a “loss”), are disruptive to emotional well-being [15]. We found that more changes increased the emotional difficulties felt by OSC. Emotional difficulties reflect the degree to which a child is worried, relates poorly to peers, is afraid, and is distracted, among other difficulties. Aside from the immediate negative feelings of fear, worry, distraction, etc., emotional well-being is important for later outcomes including educational achievement and adult functioning [31, 32]. The conditions that create a disproportionate orphan population (AIDS, war, poverty, other crises) also likely contribute to well-being. Caregivers in LMICs report substantial burden when they lack emotional and economic resources to support additional children and adolescents, which can create added household tensions and difficulties [33–35].

While we have previously found high incidence of abuse in this population [9] we did not find notable associations between instability (changes) in the familial adult composition and incident abuse. It is possible that some changes the familial adult household composition remove the threat of abuse, nullifying overall associations. It is also possible that abuse is not associated with changes in the adult household composition at the individual or population level. We also did not find associations with cognitive functioning. It is possible that cognitive functioning is generally robust to changes in familial composition changes. The Market List is advantageous in its culturally adaptive features, but may not have been sufficiently sensitive to detect change at this resolution. It may be that some amount of change is unavoidable, but additional research into which changes are particularly detrimental, and whether there are mechanisms for creating stabilizing supports, may indicate opportunities for intervention. We did not find evidence of heterogeneity by gender in any of the associations; this is consistent with our prior work in this population in which we observed, for example, that boys and girls are experiencing trauma (including abuse) at similar rates [36].

### Strengths and Limitations

This study had several notable strengths, including longitudinal follow-up spanning through adolescence of a hard-to-reach, vulnerable population in LMICs. The relevance of our conclusions is strengthened by the random sampling design of the original POFO cohort. Further, study outcomes were assessed using validated, culturally tailored measures [17–19, 37–39]. The Market List to assess cognitive functioning was administered by well-trained interviewers, though the SDQ and LEC were strictly self-report. However, both have shown good psychometric properties and have been robust in low-resource settings and across cultures [17–19, 38–41], and POFO interviewers spoke the native language of participants and made substantial efforts to gain rapport over follow-up visits to ensure honest answers. Incident abuse was measured with a combined outcome for sexual or physical abuse. For consistency with prior analyses in this population [8, 9, 36], we focused on whether any abuse was associated with changes in the familial adult household composition, though specific types of abuse may have more pronounced associations.

Potential biases included confounding and informative missing data, including loss to follow-up. We controlled for several predictors of the outcomes as well as potential confounders to mitigate potential confounding bias. We did not have an individual-level measure of socioeconomic status (SES) consistently measured over time to include, but do not expect substantial variation in this population. We also do not have information on potential household subsidies for supporting an orphaned child, reflecting unmeasured potential confounding. Retention for this population over follow-up was very good (84%). Nevertheless, to address possible informative loss to follow-up, we used robust data augmentation methods to impute incomplete observations and missed visits, pooling standard errors across imputation sets. With this approach, we mitigated the potential bias in conducting a complete case analysis, improving validity of our estimates.

Our exposure metric is limited; survey questions did not directly ask about the caregiving structure but simply identified the relations with whom OSC were living. It is reasonable to assume those adults were part of the caregiving structure, but there may have been other important adult figures we could not identify, and additional changes that were unmeasured. This is particularly true for OSC living with non-relatives; important adults in their household could have shifted, but we could not observe those changes. Our analysis assumes equal weight to the available relations because we do not know which relation may be more or less important to any given child. The importance of different relations may be particularly variable in an orphan population. We focused our analysis on number of changes in the familial adult household composition as our metric of stability; as such, we did not assign values to “gains” and “losses.” Furthermore, change could be positive or negative in nature; we could not assess the nature of the change, simply the change itself. We examined changes over a long time period; it may be that specific developmental windows are more critical than others. While change in the familial composition has been studied in more resourced countries such as the U.S. [13–15], it has not been examined in family-based care of orphans in LMICs. These results reflect average estimates across several LMICs in South Asia and East Africa, and may not be generalizable elsewhere.

### Conclusion

Ensuring orphans have care that enables them to become healthy, productive adults is a challenging endeavor, but one that is supported by the Sustainable Development Goals which call on governments to promote health and wellbeing to achieve equity and thriving societies [3]. Stability in the adult household composition is considered important to quality care, but whether stability is present for OSC in family-based care in LMICs has not been examined. To our knowledge, this study is the first to describe the familial adult household composition for family-based OSC across multiple LMICs, and the first to assess whether stability in the adult household composition is related to well-being outcomes among family-based OSC in LMICs.

There are multiple potential policy implications to this study. First, family-based care is promoted as the only viable option for global orphan care, including calls to eliminate residential care as a safety net [11, 12]. A key assumption underlying these conclusions is that family-based care is stable in terms of the adult composition of the household. Yet, family-based care has not been well-characterized in LMICs. Our findings indicate that a static familial adult household composition cannot be assumed. Second, we found that many OSC live with family members, and over half with a remaining parent. We showed that the adult family composition changes over time for many OSC, and that increased numbers of changes are associated with increased emotional difficulties. Policymakers may enhance research and programs targeting improvements in OSC well-being by considering mechanisms that help stabilize the familial adult household composition or that focus on emotional resilience in the face of instability.
